# Debt vs. revenue share financing: A theoretical study on revenue share finance in a capital-constrained newsvendor

**DOI:** 10.1371/journal.pone.0329561

**Published:** 2025-08-08

**Authors:** Yufeng Song, Minghui Jiang

**Affiliations:** School of Management, Harbin Institute of Technology, Harbin, Heilongjiang Province, China; Shandong University, CHINA

## Abstract

In the capital-constrained newsvendor paradigm, revenue share financing (RSF) presents a novel yet underexplored financing approach. This theoretical study conducts a comparative analysis of two financing methods: traditional debt financing and revenue share financing. Our research findings indicate that the revenue share ratio, cost share ratio, and debt interest rate are the most critical factors influencing retailers’ decisions. This study further demonstrates that, under certain conditions, both financing strategies can yield competitive profit margins and order quantity. Additionally, we discovered that fund providers have a minimum revenue share ratio, whereas for retailers, there is a maximum revenue share ratio. These two ratios establish a bargaining interval within which an optimal revenue share ratio can be achieved if it falls within this range. Outside this range, RSF is either unattainable or impractical.

## 1. Introduction

Access to financing for small and micro enterprises has always been a global challenge. Due to the unstable asset status, low credit ratings, and insufficient collateral, traditional financing methods often prove unfriendly to these enterprises. A World Bank (2024) survey of companies in 144 countries found that 51.4% of the companies required loans, and 21.2% considered financing availability a significant obstacle to their development. This indicates that gaining sufficient financial support during the development process is a crucial issue for these enterprises and that small and medium-sized enterprises have difficulty obtaining loans. According to the fourth economic census data released by the National Bureau of Statistics in 2018, micro and small enterprises account for 98.5% of all firms. However, according to the China Financial Yearbook 2019, the balance of loans to micro and small enterprises accounts for only 32.1% of all outstanding loans. The disparity between the proportion of micro and small enterprises and their access to capital market support, such as issuance of bond financing and listing financing, is enormous. Despite relying on informal loans from family and friends and other private lending channels, they still struggle to secure stable funding, underscoring the substantial financing difficulties faced by small and medium-sized enterprises (SMEs) in China.

In recent years, many innovative financing products and business models have emerged, and different financing methods have greatly facilitated the growth of SMEs and the market’s prosperity. The development of SMEs finance has also become a new direction for expanding business models in the financial field, attracting significant attention and research from academia and industry alike [1–6].

A new financing practice for SMEs, called revenue share financing (RSF), has recently emerged in mainland China. Unlike traditional financing, which relies on equity and debt financing—equity based on profits and debt on assets—RSF focuses on a company’s revenue and cash flow. This approach aligns with the operating cash flow reported in the company’s cash flow statement. For instance, Micro Connect, a financial company that leverages financial technology to connect global capital with small and micro enterprises in China, is pioneering this innovative financing model. The RSF offered by Micro Connect involves the investor contributing a certain amount of capital to the physical store. Within the agreed-upon timeframe, a predetermined percentage of the store’s revenue is allocated to the investor daily as a return on investment. Once this revenue share period expires, the revenue share concludes, and both parties complete the capital settlement, thus ending the partnership.

Revenue share financing is distinct from both debt and equity financing. It operates through a revenue-aligned repayment structure, where businesses repay investors a fixed percentage of monthly revenue until reaching a predetermined return cap or time deadline, avoiding equity dilution, collateral requirements, and fixed repayment schedules. Distinct from debt financing, Revenue share financing eliminates interest rates and collateral obligations, while differing from equity funding by preserving ownership control and bypassing exit mandates. Its flexibility suits businesses with stable recurring revenue—such as SaaS, subscription models, or high-margin ventures—particularly asset-light or growth-stage companies that have achieved product-market fit but lack profitability. Compared to traditional financing, Revenue share financing accelerates funding timelines and shifts risk-sharing to revenue outcomes rather than fixed obligations. As a new way of financing for SMEs, it has received mixed reviews in society: Revenue Sharing Financing allows businesses to retain ownership without collateral, offers flexible repayments tied to revenue fluctuations, and has faster approval processes than traditional financing. However, it carries higher costs than bank loans, with total fees potentially rising sharply if revenue surges, and agreements often include restrictive clauses. Its applicability is limited to non-sensitive industries with stable revenue, while startups or unprofitable ventures struggle to qualify. Additionally, the industry faces legal and regulatory compliance challenges, requiring vigilance regarding contract terms and evolving oversight risks [7,8]. However, as a novel finance business model, it can quickly develop within the current legal framework, and its underlying operation logic is worth further research.

This paper presents models integrating the two financing alternatives within the operational decisions of a newsvendor-like retailer. The model encompasses a singular capital-constrained retailer and a fund provider. The capital-constrained retailer pursues short-term financing to buy inventory from its upstream manufacturer. The retailer faces uncertain demand and operates as a newsvendor. The funding provider presents two financing options: debt financing and revenue share financing. It derives the corresponding order quantity and profitability for each financing method and subsequently deliberates on the optimal financing strategy for the retailer.

When selecting between revenue share and debt financing, critical factors include the interest rates offered by fund providers, revenue share ratio, and cost share ratio. Retailers are inclined towards RSF when debt interest rates are high. An equilibrium threshold for the revenue share ratio exists, below which retailers favor RSF; above this threshold, they opt for debt financing. A similar equilibrium threshold applies to the cost share ratio. With a fixed financing amount, fund providers have a minimum acceptable revenue share ratio; no funds will be provided below this threshold. Retailers also have a threshold for the revenue share ratio; if it exceeds this threshold, they will choose debt financing. The Nash bargaining model was utilized to optimize and compare the revenue share ratio with the thresholds, highlighting a bounded range for the revenue share ratio.

The structure of this article is as follows: Section 2 reviews relevant literature and highlights previous research. Section 3 presents a detailed model that analyzes the financing logic for both debt financing and RSF. Section 4 validates these findings through numerical analysis. Finally, Section 5 summarizes the main conclusions and suggests directions for future research.

## 2. Literature review

This article addresses supply chain contracts and the capital-constrained newsvendor model. Numerous scholars have examined the interaction between operational and financial decisions. Our research focuses on how capital constraints and financial choices influence fund providers and retailers. It draws from three main areas of literature: research on supply chain management contracts, studies of capital-constrained newsvendors and supply chain finance.

Supply chain contracts are a crucial topic in supply chain management. Previous research has extensively explored the mutual effects of different contracts in supply chains and how to optimize supply chain management using various contracts. These contracts encompass wholesale price contracts, revenue share contracts, return contracts, two-part tariff contracts, service-level agreements, quantity flexibility contracts, and so on. Cachon [9] examined six different contracts in the supply chain. He argued that compared to pure wholesale price contracts, each type of contract can induce retailers to increase their order quantities. However, no single contract universally dominates the others in all circumstances. Pasternack [7] studied return contracts in a multi-distributor environment. He found that a policy limiting returns to a fixed percentage of the purchase amount does not necessarily maximize channel profit. Instead, a return policy allowing unlimited returns of a portion of the credit can achieve this goal. Cachon and Lariviere [10] found that revenue share contracts can coordinate supply chains and allocate profits arbitrarily in the case of a single retailer. Furthermore, revenue share contracts can also coordinate supply chains in the presence of quantity competition among retailers. Wang and Pan [11] studied the coordination problem of a c contract in the presence of unreliable supply. The results show that supply chain coordination can be achieved under the quantity flexibility contract when the wholesale price and procurement commitments meet certain conditions. Wong, Qi [12] found that perfect coordination in the supply chain can be achieved when sales rebate contracts are combined with a vendor-managed inventory mechanism. This means that even when considering the retailer’s interests, they can make pricing decisions to maximize the profit of the entire supply chain. In addition, the intensity of competition also affects the profit distribution between suppliers and retailers. When competition is intense, more profit will be allocated to the manufacturer. Nie and Du [13] studied the impact of peer-induced fairness and distribution fairness on the decision-making process in a dual-level supply chain when using quantity discount contracts between suppliers and retailers. They found that quantity discount contracts cannot coordinate the supply chain when participants in the supply chain do not have fair concerns. However, by making simple adjustments to the contracts, it is possible to achieve coordination in the supply chain by ensuring that the total profit of suppliers and retailers is equal to the profit of the coordinating channel. Du, Nie [14] used the Nash game model to study a bilateral supply chain where both suppliers and retailers have concerns about fairness. They analyzed the impact of fairness concerns on optimal decisions and channel efficiency. When fairness concerns are present in the supply chain, its efficiency decreases. When suppliers are less concerned about fairness, retailers’ order quantities will be more significant. Moreover, fairness concerns do not change the state of supply chain coordination. Our research examines the strategic application of revenue share contracts, considering them as tools for supply chain coordination and mechanisms for addressing capital shortages. We analyze how the financial benefits and risks linked to revenue share contracts can impact supply chain decisions in the context of constrained capital. This approach provides a novel perspective on the intersection of finance and supply chain management.

In the study of supply chain management and inventory control, the Newsvendor Model, a classic inventory decision framework, has been widely applied across various industries. However, in reality, businesses often face capital constraints, which profoundly impact the inventory decision-making process. The capital-constrained newsvendor model has emerged to address this practical issue. Dada and Hu [15] study the inventory problem of capital-constrained newsvendors using the Stackelberg game model. It first analyzes the optimal ordering quantity for newsvendors under different interest rates. Then, from the banks’ perspective, it considers how to set the interest rates to maximize their expected profit. Through model analysis, the paper proposes a nonlinear loan plan to achieve supply chain coordination. The study shows that under an appropriate loan plan, efficient supply chain operation can be achieved, enhancing the overall system’s benefits. Kouvelis and Zhao [16] investigate the impact of supplier financing and bank financing on newsvendors and the optimal structure of trade credit contracts. The study analyzes the optimal decisions of suppliers and retailers under different financing conditions. It suggests that when the supplier’s interest rate is lower than the risk-free rate, any contract parameters that make the wholesale price equal to the optimal wholesale price under the risk-free rate are optimal. Retailers are inclined to choose supplier financing. Zhang, Wang [17] present a constrained-budget newsvendor problem with substitutable products: the newsvendor sells two substitutable products and determines the optimal order quantity and selling price for each product. The study finds that the higher the substitutability between products, the higher the retailer can set the price to achieve a more significant profit. The paper also discusses the negative impact of price wars on supply chain management. If the budget constraint is tight, the order quantity may be harmful, meaning the retailer must cancel the corresponding orders. Cheng, Wu [18] study how preferential credit based on retailer credit limits affects the operational decisions of capital-constrained newsvendors. It examines the impact of bank financing, trade credit, and combined credit on retailer inventory decisions. The study derives the equilibrium wholesale prices, expected selling prices, and order quantities for retailers under different collateral conditions and institutional risk preferences. Shi, Du [19] studied the capital-constrained newsvendor problem, where manufacturers provide repurchase contracts to compensate lenders when retailers default. The paper examines the equilibria of financing under both monopoly and competitive banking markets and analyzes the coordination strategies of the financing system. The study finds that combining repurchase contracts with wholesale price contracts can fully coordinate the entire financing system as long as the repurchase price coefficient falls within a favorable range, known as the “Pareto zone,” where all members of the supply chain system can benefit from the coordination. Shen, Wang [20] study how newsvendors can increase their loan limits by purchasing insurance in a regulated monopoly banking market. It analyzes newsvendors’ optimal order quantity and loan strategy under different initial capital and risk constraint conditions. The study finds that when the initial capital of newsvendors is low, the bank’s risk control mechanisms come into effect, resulting in the newsvendors being unable to obtain sufficient loans to achieve the optimal order quantity. In such cases, purchasing insurance can reduce the bank’s loan risk, increasing loan limits and order quantities for the newsvendors. Most existing studies concentrate on banks as fund providers, emphasizing how interest rates influence the newsvendor problem. In contrast, our research examines how RSF can mitigate funding shortages and enhance supply chain efficiency compared to traditional debt financing. We adopt a methodology similar to that of Huang, Yang [21] to find the optimal revenue share ratio.

The diversity and applicability of financing strategies in supply chain finance have become a focal point of recent research, with extensive discussions on the differences among financing methods in terms of risk-sharing, funding availability, and supply chain synergy. Existing literature on financing mode selection primarily analyzes three dimensions: debt financing, equity financing, and hybrid financing. Debt financing emphasizes high accessibility and operational efficiency. For instance, bank credit financing can rapidly alleviate corporate liquidity pressures while dispersing risk [22]. Trade credit financing, known for its low cost and flexibility, is widely adopted for short-term intra-supply-chain financing [23]. However, debt financing’s limitations lie in its accumulation of financial risks and funding constraints. For example, bank credit may exacerbate financial risks due to fixed repayment obligations [22], and credit-guaranteed financing, while expanding funding scales, increases suppliers’ risk exposure [23]. Additionally, default risks under debt financing become pronounced under demand volatility [24]. In contrast, equity financing achieves risk-sharing and long-term alignment through profit-sharing mechanisms. For example, supplier investment relieves retailers’ immediate repayment pressure via profit-sharing [25], while equity-based supplier financing converts default risks into investment risks, diversifying risks through portfolio strategies and enhancing supply chain coordination [24]. Notably, in markets with high demand potential, equity financing’s profit-sharing model is particularly attractive to risk-averse manufacturers [26]. Hybrid financing strategies aim to balance the strengths and weaknesses of both approaches. For instance, a hybrid model of bank credit and equity financing optimizes capital structures to reduce financing costs [17], while combining supplier financing with investment balances funding flexibility and risk diversification [22]. However, this comes at the cost of increased managerial complexity and coordination efforts. The applicability of financing strategies heavily depends on the financial status and risk preferences of supply chain members. For instance, equity financing is more likely to be adopted when retailers face severe capital constraints due to its risk-sharing nature [26], whereas debt financing is favored when capital is relatively sufficient or production costs are low, owing to its stable return structure. In terms of default risk mitigation, equity financing’s long-term alignment mechanism significantly outperforms pure debt financing [24]. In summary, existing studies suggest that financing strategy selection requires balancing funding availability, risk distribution and management costs. The rise of hybrid financing reflects an evolution from single-mode solutions to structured approaches, but its effective implementation relies on supply chain transparency and optimized contract design. While existing supply chain finance literature predominantly focuses on debt financing, equity financing, and hybrid financing, revenue share financing remains underexplored. This study systematically investigates revenue share financing, revealing threshold effects in financing mode selection within supply chain finance. Furthermore, through game-theoretic modeling, we rigorously derive the optimal revenue share ratio, offering novel insights into this emerging paradigm.

## 3. Model

This section focuses on studying the capital-constrained newsvendor problem involving two parties: a capital-constrained retailer and a fund provider. The retailer requires short-term financing to purchase a single product as an inventory, which will be sold to consumers. The primary objective of this section is to explore the interactions among these two parties within the framework of the newsvendor model. We consider two financing methods: debt financing and the RSF.

In the first stage, the wholesale price w is fixed, and then the retailer determines the purchasing quantity q based on this price. The initial capital of the retailer is x, and if the purchasing cost wq exceeds x, the fund provider can supply the additional funds. This article explores two methods: debt financing and RSF. Under debt financing, the fund provider provides the required funds in exchange for repayment and interest in the second stage. In contrast, with RSF, the fund provider supplies the necessary funds but receives a predetermined percentage of the revenue as financing costs based on actual sales in the second stage. Regardless of the financing method chosen, the retailer makes the initial product purchase in the first stage, and in the second stage, market demand is realized, prompting the retailer to reimburse the fund provider according to the selected financing arrangement.

### 3.1. Notation and assumptions

The retailer’s decision includes the purchasing quantity q and the choice of financing method. The exogenous parameters are the risk-free rate $r_{f},\;$debt interest rate $r_{D},\;$(where $r_{D}\geq r_{f})$, the unit retail price p, and wholesale price w. To simplify the model without losing generality, we exclude the salvage value of unsold items and the associated goodwill loss, as they do not fundamentally alter the problem. To avoid trivial scenarios, we assume $p>(1+r_{D})w>0$. The market demand, denoted by d is uncertain. Let F(·) be the cumulative distribution function and f(·) be the probability density function of d. We assume F(d) is a strictly increasing and differentiable function, F(0)=0, and E(d) denote the expected demand. These assumptions are aligned with those presented by Kouvelis and Zhao (16) in their study. Furthermore, all participants in this model are considered trustworthy and are expected to adhere to the contractual agreements. Table 1. list all the notations used in this model.

**Table 1:** Notations of the Models.

Notation	Descriptions	Notation	Descriptions
d	Market demand	θ	Revenue share ratio 0<θ≤1
x	Retailer’s initial capital	δ	Cost share ratio 0<δ≤1
q	Retailer’s order quantity	$\pi_{R}$	Retailer’s profit when no financing need
w	Wholesale price	$\pi_{R}^{D}$	Retailer’s profit under debt financing
p	Market price	$\pi_{R}^{M}$	Retailer’s profit under RSF
$r_{f}$	Risk-free rate	$\pi_{I}^{D}$	Fund provider’s profit under debt financing
$r_{D}$	Debt interest rat	$\pi_{I}^{M}$	Fund provider’s profit under RSF

### 3.2. Model analysis

If the retailer has sufficient funds in the first stage, the profit can be expressed as follows:


$$\pi_{R}=E[min(d,q)]p-wq+(x-wq)(1+r_{f})\;$$ (1)

Here, the term reflects the investment income earned at the risk-free interest rate from the retailer’s additional funds, after accounting for the necessary capital for purchasing. The optimal order quantity is given by $q_{1}^{*}=F^{-1}\left(1-w(1+r_{f})/p\right)$. When the retailer’s capital is adequate, the determination of the optimal order quantity is influenced by various factors, including the product’s probability distribution, market price, wholesale cost, and risk-free interest rate. This formulation embodies the critical fractile solution for optimal inventory decisions in the classical newsvendor model.

#### 3.2.1. Debt financing.

When the retailer faces a shortage of funds and opts to use debt financing, three distinct scenarios need to be considered:

**No initial capital:** If the retailer begins with no initial capital x=0, the profit function can be represented as:


$$\pi_{R}^{B}=E[min(d,q)]p-wq\left(1+r_{D}\right)$$ (2)

In this case, the optimal order quantity in this situation is $q_{2}^{*}=F^{-1}\left(1-w(1+r_{D})/p\right)$.

**Using all capital:** If the retailer first exhausts all initial capital and then borrows from the fund provider, the profit function is represented as:


$$\pi_{R}^{B}=E[min(d,q)]p-(wq-x)\left(1+r_{D}\right)-x$$ (3)

The optimal order quantity in this situation is $q_{2}^{*}=F^{-1}\left(1-w(1+r_{D})/p\right)$*.*

**Borrowing while retaining some Capital for Investment:** If the retailer chooses to borrow from the fund provider while retaining some initial capital for investment, generating returns at the risk-free rate, the profit function can be expressed as:


$$\pi_{R}^{B}=E[min(d,q)]p-(1-\delta )wq+(x-(1-\delta )wq)\;r_{f}-\delta wq(1+r_{D})$$ (4)

In this scenario, *δ* represent the cost share ratio indicating the percentage of expenses covered by the fund provider. The optimal order quantity is $q_{2}^{*}=F^{-1}\left(1-w(1-\delta r_{f}+r_{f}+\delta r_{D})/p\right)$*.*

After analyzing three distinct financial scenarios, we find that the optimal order quantity remains unchanged when a retailer uses all available funds—regardless of whether they come from initial capital or other sources. However, when the retailer secures a debt while reserving some funds for alternative uses, the optimal order quantity increases relative to scenarios where all funds are exclusively allocated to purchasing.

From the perspective of the fund provider, there is a risk of loss if the retailer fails to generate sufficient revenue to meet loan obligations. If the retailer cannot repay the loan with interest, the retailer will go bankrupt. We denote the demand threshold as $\hat{d}$ where $\hat{d}=(wq-x)\left(1+r_{D}\right)/p$. If the actual demand d falls below $\hat{d}$, the retailer will go bankrupt. Furthermore, we introduce another threshold $\overset{―}{d}$, defined as *.* $\overset{―}{d}$ represent the profit for the retailer with financing and without financing are the same. In cases where demand falls within the range $d\in \left(\hat{d},\overset{―}{d}\right)$, the retailer will be able to repay the loan, but the remaining profit will be less than the revenue that would have been earned without financing. Hence, the fund provider’s profit can be represented as:


$$\pi_{I}^{D}=\delta wqr_{D}-p\int_{0}^{\hat{d}}F(x)dx$$ (5)

#### 3.2.2. Revenue share financing.

Now consider the case where the retailer chooses the RSF. If a retailer experiences a financial shortage and chooses RSF to acquire funds, there also are three scenarios that also need to be considered:

**No initial capital**: If the retailer does not have any initial capital *x = 0*, the profit function can be represented as follows:


$$\pi_{R}^{M}=E[min(d,q)](1-\theta )p$$ (6)

Where θ∈[0,1] represents the proportion of total revenue taken by the fund provider under the RSF model. The optimal order quantity in this situation is $q_{3}^{*}=F^{-1}(1)$*.* This indicates that when all capital is derived solely from the fund provider, the retailer will opt to procure the maximum possible order quantity.

**Using all capital**: If the retailer first spends all his initial capital and then uses the RSF to fulfill the capital need with no retaining, the profit function can be represented as follows:


$$\pi_{R}^{M}=E[min(d,q)](1-\theta )p-(1-\delta )wq$$ (7)

where δ∈[0,1] represents the proportion of inventory costs borne by the fund provider. The optimal order quantity in this situation is $q_{3}^{*}=F^{-1}\left(1-(1-\delta )w/p(1-\theta )\right)$*.*

**Borrowing while retaining some capital for investment:** Suppose the retailer chooses RSF and retains a portion of his initial capital to invest and gain a return equal to the risk-free return. Under this scenario, the profit function can be represented as follows:


$$\pi_{R}^{M}=E[min(d,q)](1-\theta )p-(1-\delta )wq+(x-(1-\delta )wq)r_{f}$$ (8)

where x−(1−δ)wq represent when the initial capital of the retailer exceeds the inventory cost, the retailer will invest the remaining funds and receive a return equal to the risk-free rate. The optimal order quantity in this situation is $q_{3}^{*}=F^{-1}\left(1-\left(1-\delta -\delta r_{f}+r_{f}\right)w/p(1-\theta )\right)$*.*

Through the above analysis, we found that in the case where the initial capital of the retailer is 0, the optimal order quantity is equal to the maximum market capacity. In this scenario, the absence of cost input compels the retailer to pursue an aggressive strategy to capture the entire market share to maximize profits. However, this approach may impose more significant costs on the fund provider, potentially leading to capital losses. As a result, under these conditions, the fund provider may be reluctant to extend credit. Moreover, our analysis reveals that the optimal order quantity is greater when the retailer operates without surplus funds as compared to situations where surplus funds are maintained.

As for the fund provider, in the RSF, he also needs to bear the risk of loss if the retailer fails to generate sufficient revenue. However, the retailer is not exposed to bankruptcy risks, as they will always retain a certain amount of funds at the close of the transaction: E[min(d,q)](1−θ)p*.* We define the demand threshold as $\hat{d}$ where $\hat{d}=(1-\delta )wq/\theta p$*.* If the actual demand $d<\hat{d}$, the retailer will repay less than δwq resulting in a loss for the fund provider. Another threshold is denoted as $\overset{―}{d}$, is defined by $\overset{―}{d}=wq/p$. If the actual demand falls within the interval $d\in \left(\hat{d},\overset{―}{d}\right)$, the retailer can repay more than δwq, however, the remaining capital will be less than what would have been available without financing. The fund provider’s profit can be represented as:


$$\pi_{I}^{M}=E[min(d,q)thetap-p\int_{0}^{\hat{d}}F(x)dx$$ (9)

To analyze the attractiveness of different financing methods for fund providers, we first consider the expected income generated from investments in the RSF model, represented as E[min(d,q)]θp, and income derived from debt financing as $\delta wqr_{B}$. Additionally, the potential loss incurred can be quantified as $p\int_{0}^{\hat{d}}F(x)dx$. For these two financing methods to be perceived as equally attractive to fund providers, their respective revenue must match $\pi_{I}^{M}=\pi_{I}^{D}$. It is important to note that each financing method establishes distinct demand thresholds for fund providers. In RSF, this threshold is defined as $\;{\hat{d}}_{RSF}=(1-\delta )wq/\theta p$ while the threshold in debt financing is ${\hat{d}}_{debt}=(wq-x)\left(1+r_{D}\right)/p$. these two thresholds converge when $\theta =1/(1+r_{D})$. In this scenario, the income for fund providers in both models are equal: $E[min(d,q)]\theta p=\delta wqr_{B}$. Conversely, if $\theta <1/(1+r_{D})$, it results in $E[min(d,q)]\theta p>\delta wqr_{D}$, indicating fund provider need to take away more capital from the retailer under RSF. This is because, under the RSF model, fund providers must bear a more significant risk level than debt financing.

### 3.3. The uniform distribution scenario comparison analysis

In the above, we analyzed retailers’ profit and optimal order quantity under three different scenarios. In this section, we focus on the case in which the retailer’s initial capital and financing funds equals the purchasing cost. To compare two financing models, we assume that the uncertainty of demand d follows a uniform distribution U[0, u]. The optimal order quantity for the debt financing model is given by $q_{2}^{*}=u\left(1-w(1+r_{D})/p\right)$, while for the RSF model, it is given by $q_{3}^{*}=u\left(1-(1-\delta )w/p(1-\theta )\right)$.

Let ΔQ denote the difference between the optimal order quantities in the two models. This difference can be expressed as:


$$\Delta Q=q_{2}^{*}-q_{3}^{*}=\frac{uw\left((1-\theta )r_{D}+\delta -\theta \right)}{(\theta -1)p}$$ (10)

**Corollary 1.** The difference in optimal order quantity has the following properties:$\frac{\partial \Delta Q}{\partial \delta }<0\;,\frac{\partial \Delta Q}{\partial \theta }>0,\;and\;\frac{\partial \Delta Q}{\partial r_{D}}<0$.

According to Corollary 1, the cost share ratio and the interest rate exert a negative impact on ΔQ. In contrast, the revenue share ratio has a positive effect on ΔQ. When $\delta >\theta -(1-\theta )r_{B},\Delta Q>0$, indicating that the optimal order quantity under the debt financing model exceeds that under the RSF model. Conversely, when $\delta <\theta -(1-\theta )r_{D}$, ΔQ<0, meaning that the optimal order quantity under the RSF model exceeds that under the debt financing model, retailers aiming to increase their order quantities should consider these findings.

When all available capital is invested in purchasing, and the uncertainty of demand d follows a uniform distribution U[0, u]. Incorporating the optimal order quantity into each profit function, the profits for the retailer in the different models are given by:


$$\pi_{R}^{B}=\frac{r_{D}(uw^{2}r_{B}+2uw(w-p)+2px)+u{\left(p-w\right)}^{2}}{2p}$$ (11)

$$\pi_{R}^{M}=\frac{u{\left((\theta -1)p-\delta w+w\right)}^{2}}{2(1-\theta )p}$$ (12)

Let $\Delta \pi {=\pi }_{R}^{D}-\pi_{R}^{M}$ represent the profit difference between the two models:


$$\Delta \pi =\frac{(\theta -1)r_{D}\left(uw^{2}r_{D}+2uw(w-p)+2px\right)+u\left((\theta -1)\theta p^{2}-2\delta (\theta -1)pw+w^{2}\left((\delta -2)\delta +\theta \right)\right)}{2(\theta -1)p}$$ (13)

**Proposition 1.** *F*or the retailer, there exists** $\hat{\delta }$*. in which the difference in profits between the two financing models is equal to zero* Δπ=0*.When* $\delta <\hat{\delta }$ *the retailer will choose the debt model for financing. Conversely, when* $\delta >\hat{\delta }$ *the retailer will choose the RSF.*

Proposition 1 indicates that the retailer’s financing choice is affected by the cost share ratio. When the cost share ratio is relatively high $\delta >\hat{\delta }$, the retailer will prefer the RSF to maximize profits. This preference stems from the fact that a higher cost share ratio transfers more market risk from the retailer to the fund provider, thereby reducing the initial capital required from the retailer. When the cost share is relatively low $\delta <\hat{\delta }$, the retailer will opt for debt financing, resulting in higher profits.

**Proposition 2.** *For the *retailer, there exists** $\hat{\theta }$ *which the profit differential between the two financing models is also zero* Δπ=0*. When* $\theta >\hat{\theta }$*, the retailer will choose the debt model financing. When* $\theta <\hat{\theta }$*, the retailer will choose the RSF.*

In debt financing, the cost of funds is fixed at a specific interest rate. In contrast, the cost of funds in RSF varies according to the revenue share ratio. As Proposition 2 indicates, when the revenue share ratio is relatively high $\theta >\hat{\theta }$, the cost of funds increases, leading the retailer to prefer debt financing. Conversely, when the revenue share ratio is relatively low $\theta <\hat{\theta }$, enabling the retailer to attain higher profits by opting for RSF.

**Corollary 2.** The difference in profit for the retailer under two different financing methods has the following quantities:$\frac{\partial \Delta \pi }{\partial \delta }<0\;and\;\frac{\partial \Delta \pi }{\partial \theta }>0$

Corollary 2 indicates that the revenue and cost share ratios significantly influence the retailer’s profit difference. Specifically, an increase in the revenue share ratio leads the retailer to favor debt financing. In contrast, an increase in the cost share ratio enhances the attractiveness of the RSF option for the retailer.

### 3.4. The Normal Distribution Scenario Analysis

While uniform distribution provides a tractable framework for comparing financing strategies, real-world demand patterns often exhibit characteristics better captured by the normal distribution. Under the assumption that demand follows a normal distribution $d\sim N(\mu ,\sigma^{2})$, the expected sales can be written as:


$$E\left[\min(d,q)\right]=\mu \Phi \left(\frac{q-\mu }{\sigma }\right)+q\left(1-\Phi \left(\frac{q-\mu }{\sigma }\right)\right)-\sigma \phi \left(\frac{q-\mu }{\sigma }\right)$$ (14)

Where Φ(·) and ϕ(·) denote the cumulative distribution function and probability density function of the standard normal distribution, respectively.

The profit function under debt financing can be represented as follows:


$$\pi_{R}^{B}=\mu \Phi \left(\frac{q-\mu }{\sigma }\right)+q\left(1-\Phi \left(\frac{q-\mu }{\sigma }\right)\right)-\sigma \phi \left(\frac{q-\mu }{\sigma }\right)-(wq-x)\left(1+r_{D}\right)-x$$ (15)

Then the optimal order quantity satisfies the following equation:


$$\Phi \left(\frac{q^{*}-\mu }{\sigma }\right)=1-\frac{\left(1+r_{D}\right)w}{p}$$ (16)

Further analysis of the sensitivity of $q^{*}$ to demand volatility σ using the implicit function theorem shows:


$$\frac{\partial q^{*}}{\partial \sigma }=\frac{q^{*}-\mu }{\sigma }$$ (17)

**Proposition 3.** *U*nder debt financing, the impact of demand volatility* σ *on the optimal order quantity** $q^{*}$ *follows these patterns:*

*When profits are high and* $q^{*}>\mu$*, the sensitivity of* $q^{*}$ *to* σ *is positive:* $\frac{\partial q^{*}}{\partial \sigma }>0$*. When profits are low and* $q^{*}<\mu$*, the sensitivity of* $q^{*}$ *to* σ *is negative:* $\frac{\partial q^{*}}{\partial \sigma }<0$*.*

When the retail price p is significantly higher than the wholesale price w, retailers tend to increase order quantities to capture upside demand gains $\frac{\partial q^{*}}{\partial \sigma }>0$. The high marginal profit makes stockout costs much higher than overstocking risks. The fixed interest rate of debt financing amplifies profit advantages through leverage, encouraging aggressive strategies.

When p is close to w, increased demand volatility σ leads retailers to reduce order quantities $\frac{\partial q^{*}}{\partial \sigma }<0$. Here, the financial risk of overstocking dominates decisions. Financing cost pressures squeeze profit space, pushing retailers toward conservative strategies to avoid debt repayment crises.

These dynamics reveal a corporate trade-off mechanism: retailers “bet on demand increases” when profits are high and “guard against demand decreases” when profits are low. The interplay of debt costs and volatility shapes optimal inventory strategies. Notably, under revenue-sharing financing, despite different risk-sharing mechanisms, the consistent sensitivity direction indicates that retailers’ responses to demand volatility are universal. This further confirms the model’s robustness.

### Financing cost analysis

In the bank financing model, the financing cost is expressed as:


$$C_{B}=r(wq-x)$$ (18)

Under the normal distribution, substituting the optimal order quantity: $q^{*}=\mu +\sigma \Phi^{-1}(1-\frac{(1+r)w}{p})$ the cost becomes:


$$C_{B}=r\left(w\mu +w\sigma \Phi^{-1}\left(1-\frac{(1+r)w}{p}\right)-x\right)$$ (19)

The sensitivity to demand volatility σ is:


$$\frac{\partial C_{B}}{\partial \sigma }=rw\Phi^{-1}\left(1-\frac{(1+r)w}{p}\right)>0$$ (20)

This indicating higher volatility increases financing costs due to the fixed interest structure amplifying borrowing needs.

Under RSF, the financing cost is defined as:


$$C_{R}=\theta pE\left[\min(d,q)\right]$$ (21)

Under the normal distribution, substituting the optimal order quantity $q^{*}=\mu +\sigma \Phi^{-1}(1-\frac{(1-\delta )w}{(1-\theta )p})$, the cost becomes:


$$C_{R}=\theta p\mu +\frac{\theta (1-\delta )\sigma w}{1-\theta }\Phi^{-1}(z)-\theta p\sigma \varphi (z)$$ (22)

where $z=\Phi^{-1}(1-\frac{(1-\delta )w}{(1-\theta )p})$.

The sensitivity to σ is:


$$\frac{\partial C_{R}}{\partial \sigma }=\theta p\left(z\left(1-\Phi (z)\right)-\varphi (z)\right)<0$$ (23)

This reveals that higher volatility reduces financing costs. This counterintuitive result stems from RSF’s risk-sharing mechanism: the fund provider internalizes demand volatility through revenue and cost-sharing ratios, thereby mitigating the retailer’s financial burden.

**Proposition 4.** *Under bank financing and RSF, the sensitivity of financing costs to market volatility* σ *exhibits significant divergence:*


$$\frac{\partial C_{B}}{\partial \sigma }=rw\Phi^{-1}\left(1-\frac{(1+r)w}{p}\right)>0$$

$$\frac{\partial C_{R}}{\partial \sigma }=\theta p\left(z\left(1-\Phi (z)\right)-\varphi (z)\right)<0$$

Under bank financing, financing costs exhibit a positive sensitivity to market volatility. This arises because heightened demand volatility incentivizes retailers to increase their optimal order quantity, amplifying borrowing needs to finance larger inventory purchases. The fixed interest rate, applied proportionally to the loan amount, translates this expanded borrowing into higher financing costs. In contrast, RSF demonstrates an inverse relationship, with financing costs decreasing as volatility rises.

Two mechanisms drive this counterintuitive result: first, the risk-sharing mechanism inherent in RSF redistributes demand volatility risks between the retailer and fund provider through negotiated revenue and cost shares, effectively internalizing a portion of the uncertainty; second, the revenue compensation effect leverages higher expected sales revenue under volatility to offset proportional repayment obligations. This dynamic highlights RSF’s adaptability to volatile markets, where flexible risk allocation mitigates financial strain, whereas bank financing—with its rigid interest structure—remains better suited to stable demand environments.

### 3.5. Revenue share ratio bargaining

We employ the Nash bargaining model to explore the fund provider’s alternatives, assuming that the fund provider and the retailer can negotiate to establish the revenue share ratio within the RSF framework, the objective is to identify a mutually acceptable ratio for both parties.

Let Q=E[min(d,q)]p represent the expected revenue of the retailer, $g=p\int_{0}^{\hat{d}}F(x)dx$ represent the potential loss of the fund provider, B represent the retailer borrowing amount, $\pi_{w}^{R}=(p-w)(x/w)$ represent the retailer’s profit without financing. Additionally, $\;T=\pi_{I}^{M}+\pi_{R}^{M}$ represents the total profit generated if cooperation occurs. Without RSF, the fund provider’s profit will be zero. The Nash bargaining model aims to identify the optimal point where both the retailer and the fund provider maximize their mutual benefits. The bargaining game under RSF can be expressed as follows:


$$\max\left(\pi_{I}^{M},\pi_{R}^{M}\right)=(\pi_{I}^{M})(\pi_{R}^{M}{-\pi }_{R}^{W})$$

$$s.t.\;{\;\pi }_{R}^{M}>\pi_{R}^{W},\pi_{I}^{M}>0,\;\pi_{I}^{M}+\pi_{R}^{M}=T$$

The equation can be rewritten as:


$$\left(Q\theta -B-g)(Q(1-\theta )-x-B-\pi_{R}^{W}\right)$$ (24)

**Proposition 5.** *Th*e optimal revenue share ratio is** $\theta^{*}=(Q+g-x{-\pi }_{R}^{W})/2Q$*, At this ratio, both the retailer and the fund provider can achieve a Nash equilibrium that maximizes their respective profits.*

The above analysis determines the optimal revenue share ratio. However, bargaining is not feasible in a perfectly competitive capital market, and the fund provider cannot arbitrarily set the revenue share ratio. Instead, the fund provider’s total returns remain consistent, and long-term profits generally approach zero. Under these conditions, the profit function for the fund provider in a revenue sharing scenario can be expressed as follows:


$$\pi_{I}^{M}=Q\theta -B-g=0$$ (25)

In this scenario, the revenue share ratio is fixed at $\overset{―}{\theta }=(B+g)/Q$*.* This ratio, $\overset{―}{\theta }$ can be considered the fund provider’s minimum acceptable threshold. If the actual revenue share ratio falls below $\overset{―}{\theta }$, the fund provider will refrain from lending to the retailer, as any ratio beneath this threshold would inevitably result in a loss for the fund provider or yield less profit compared to debt financing.

Assuming the retailer has the same expected total revenue and borrowing amount across different financing modes within the same market environment, the profit function of the retailer under debt financing can be represented as $\pi_{R}^{D}=Q-B\left(1+r_{D}\right)-x$. The profit function of the retailer under the RSF mode can be represented as $\pi_{R}^{M}=Q(1-\theta )-x$. The difference in profits between the two financing models can be calculated as:$\Delta \pi =\pi_{R}^{D}-\pi_{R}^{M}=Q\theta -B\left(1+r_{D}\right)$.

In this context, there exists a revenue share ratio $\tilde{\theta }=B\left(1+r_{D}\right)/Q$. that equates the profit difference to zero. If $\;\theta >\tilde{\theta }$, the retailer should opt for debt financing; if $\theta <\tilde{\theta }$, RSF is preferable. Consequently, a bargaining space is defined as $\theta \in (\overset{―}{\theta },\tilde{\theta })$. When $\;\overset{―}{\theta }<\theta^{*}<\tilde{\theta }$, the fund provider and retailer can reach a Nash bargaining optimal revenue share ratio if the bargaining is feasible. In the condition where $\theta^{*}>\tilde{\theta }$, the optimal ratio is not achievable, and if $\;\overset{―}{\theta }>\tilde{\theta }$, the RSF option will be unsuitable for both parties.

In conclusion, we identified three potential revenue share ratios: $\overset{―}{\theta },\tilde{\theta }\;and\;\theta^{*}$. If the fund provider has exclusive authority over determining the revenue share ratio, he will select $\tilde{\theta }$. If the ratio is negotiated between the fund provider and the retailer, $\tilde{\theta }$ will be chosen if feasible. Conversely, if the retailer possesses the authority to determine the ratio, he will opt for $\overset{―}{\theta }$.

## 4. Numerical analysis

In this section, we conducted a numerical analysis to validate our findings and examined the impact of different parameters under two financing models. The numerical parameters utilized in our study are as follows: $p=1,\;w=0.4,\;x=1,r_{D}=r_{f}=0$*.* The uncertainty of demand d follows a uniform distribution U [0, u]and u=10. This part mainly examines whether the above proposition and result are correct.

**Fig 1:**
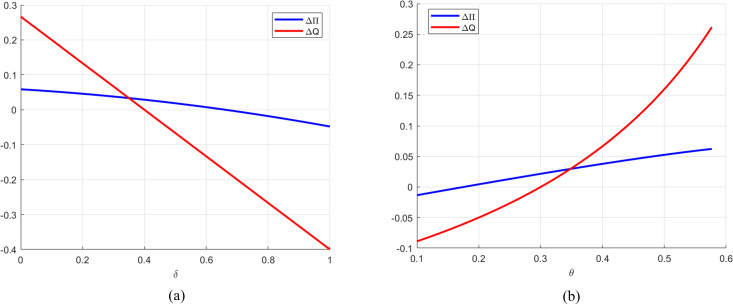
Impact of cost share ratio and revenue share ratio on Δπ and ΔQ.

The analysis of the combined data presented in Fig 1. reveals significant relationships between cost share ratio δ and revenue share ratio θ with Δπ and ΔQ. A notable negative correlation exists between δ, Δπ and ΔQ, suggesting that as δ increases, Δπ and ΔQ decrease. Specifically, when θ=0.4 and δ>0.6619, retailers should opt for RSF to maximize profits, whereas debt financing is more advantageous when δ<0.6619. Additionally, for θ=0.4 and δ>0.4, the optimal order quantity under RSF exceeds that of debt financing; conversely, when δ<0.4, the opposite holds.

Concerning θ, a positive correlation is observed with both Δπ and ΔQ. As θ increases, Δπ and ΔQ also increase. For instance, when δ=0.3 and θ>0.175, debt financing is recommended for higher profits, while RSF is advantageous when θ<0.175. Furthermore, when δ=0.3 and θ>0.3, the optimal order quantity under debt financing surpasses that under RSF, whereas it is greater for RSF when θ<0.3.

**Fig 2:**
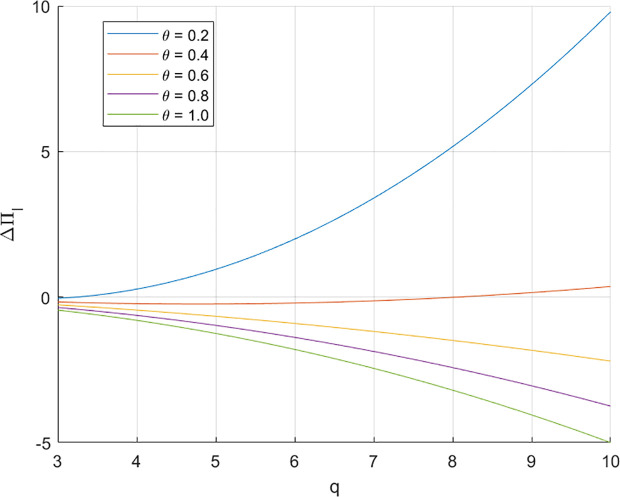
Effect of q and θ on $\Delta \pi_{I}$.

Assuming the same conditions as mentioned earlier, let $\Delta \pi_{I}$ represent the profit difference for the fund provider. Under the same assumption as above, we choose an order quantity of 3 as the starting point since the retailer must seek financing for orders of this size or larger. when  q=3, the revenue share ratio θ need to be 0.392 to achieve $\Delta \pi_{I}=0$*.* Fig 2. illustrates how varying levels of θ impact the fund provider’s profit

Assuming the same conditions as mentioned earlier, let $\Delta \pi_{I}$ represent the profit difference for the fund provider. Under the same assumption as above, we choose an order quantity of 3 as the starting point since the retailer must seek financing for orders of this size or larger. When  q=3, the revenue share ratio θ need to be 0.392 to achieve $\Delta \pi_{I}=0$. Fig 2. illustrates how varying levels of θ impact the fund provider’s profit difference under different financing amounts, as θ increase, fund provider’s profit under RSF increases.

**Fig 3:**
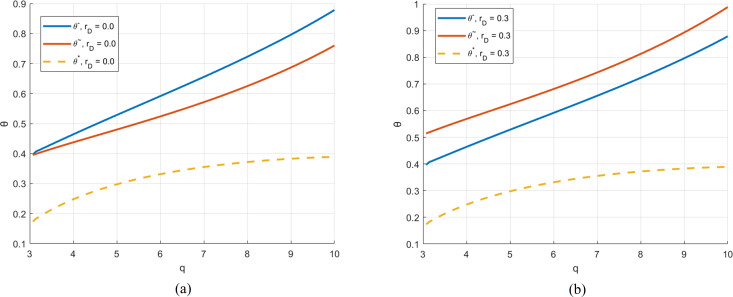
$\overset{―}{\theta }$, $\theta^{*}$ and $\tilde{\theta }$ under different $r_{D}$.

Fig 3 illustrates the impact of changes in the debt interest rate on $\overset{―}{\theta },\tilde{\theta }\;and\;\theta^{*}$. When the debt interest rate is set to zero, $\overset{―}{\theta }$ is always larger than $\;\tilde{\theta }$, indicating that RSF is not a viable option for both parties. This is because, with a zero-interest rate, borrowing use debt becomes cost-free, and the retailer will always choose the debt financing. However, when $r_{D}=0.3$ as shown on the right side of Fig 3. Because $\overset{―}{\theta }<\tilde{\theta }$, in this scenario there is a bargaining space for the revenue share ratio for both parties. The debt interest rate significantly affects the value of $\overset{―}{\theta }$. Nonetheless, in both scenarios, the optimal revenue share ratio, $\theta^{*}$ remains unachievable under any circumstances.

**Fig 4:**
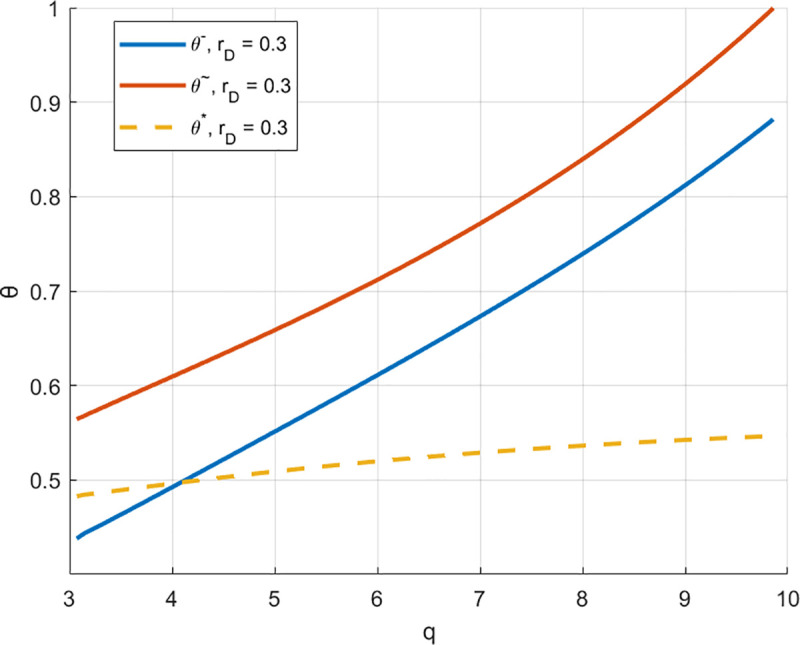
$\overset{―}{\theta }$, $\theta^{*}$ and $\tilde{\theta }\;$when $r_{D}=0.3$ and x=0.1.

Fig 4 Show the case which $\theta^{*}$ can be achieved, with $\theta^{*}$ being primarily affected by the initial capital x. As x increase, $\theta^{*}$ decrease. If the initial capital is set at x=0.1 with all other conditions held constant, the optimal revenue share ratio is between $\overset{―}{\theta }$ and $\tilde{\theta }$, therefore the optimal condition can be achieved. When q=3.51, $\theta^{*}=\overset{―}{\theta }=0.465$. If q<3.51, the optimal revenue share ratio can be attained. Conversely, when q>3.51, the optimal ratio cannot be achieved. However, RSF remains accessible regardless of the purchase quantity.

## 5. Conclusion

This study examines a financially constrained newsvendor model. First, we analyze how a retailer’s financing choice (debt or revenue share) affects profits and optimal order quantity under financial constraints. In the construction of the model, we consider three scenarios. In the first scenario, we assume the retailer has zero initial capital. In the second scenario, retailers have fully exhausted their initial and financing capital for inventory purposes. In the third scenario, retailers retain some of their funds for inventory after financing. Our analysis reveals that the optimal order quantity is the same under debt financing in the first two scenarios. In the third scenario, the optimal order quantity is influenced by factors such as the cost share ratio, debt interest rate, and risk-free interest rate. When retailers opt for RSF, in the first scenario, the retailer will try to capture all market capacity since there are no associated costs. In the second scenario, the optimal order quantity is affected by the revenue share and cost share ratio. In the third scenario, the debt interest rate and risk-free rate also influence the optimal order quantity.

We then compared the impact of two financing models on retailers’ decisions, focusing on revenue and cost share ratios under the assumption that the market is uniformly distributed. An equilibrium point $\hat{\theta }$ exists for the revenue share ratio. When $\theta <\hat{\theta }$ the retailer will choose RSF. Conversely, the retailer will choose debt financing. Similarly, an equilibrium point $\hat{\delta }$ exists for the cost share ratio; when $\delta >\hat{\delta }$, the retailer will choose RSF, and when $\delta <\hat{\delta }$, the retailer will choose debt financing.

We employed the Nash bargaining model to identify the optimal revenue share ratio. Through Nash bargaining, we identify a bounded interval for θ where mutually beneficial agreements between retailers and fund providers are achievable. Outside this range, RSF becomes impractical, emphasizing the importance of aligning contractual terms with market conditions and risk appetites. Then we analyze how demand volatility affects the financing market. Under debt financing, rising demand volatility amplifies financing costs due to rigid interest obligations. In contrast, RSF mitigates this effect through proportional risk-sharing, making it more adaptable to uncertain markets.

Examining revenue share as a financing mechanism presents considerable potential for further investigation. This study simplifies the model by analyzing a scenario with a single fund provider and a single retailer. Future research could explore how competition affects retailers’ financing decisions with multiple parties. Moreover, the revenue share model raises several compelling issues for inquiry, including potential moral hazards, adverse selection challenges faced by retailers, and risk management difficulties encountered by fund providers.

## Appendix

Proof for $q_{2}^{*}=F^{-1}\left(1-\frac{w\left(1+r_{B}\right)}{p}\right)$

We derived the optimal order quantity by differentiating the profit function under debt financing, setting the derivative to zero, solving for the critical cumulative demand value, and applying the inverse cumulative distribution function.


$$\pi_{R}^{B}=\left[q-\int_{0}^{q}F(x)dx\right]p-(wq-x)\left(1+r_{B}\right)-x$$

Set $\frac{\partial \pi_{R}^{B}}{\partial q}=p-F(q)p-w\left(1+r_{B}\right)=0$


$$q_{2}^{*}=F^{-1}\left(1-\frac{w\left(1+r_{B}\right)}{p}\right)$$

Proof for $q_{3}^{*}=F^{-1}\left(1-\frac{\left(1-\delta -\delta r_{f}+r_{f}\right)w}{p(1-\theta )}\right)$

We employed a method analogous to that used for Proposition 1 to determine the optimal order quantity under the revenue-sharing model.


$$\pi_{R}^{M}=p(1-\theta )\left[q-\int_{0}^{q}F(x)dx\right]-(1-\delta )wq+\left(x-(1-\delta )wq\right)r_{f}$$

Set $\frac{\partial \pi_{R}^{M}}{\partial q}=\left(1-F(q)\right)(1-\theta )p-(1-\delta )w-(1-\delta )wr_{f}=0$


$$q_{3}^{*}=F^{-1}\left(1-\frac{\left(1-\delta -\delta r_{f}+r_{f}\right)w}{p(1-\theta )}\right)$$

Proof of **Corollary 1.**

We compute the partial derivatives of the change in the optimal order quantity ΔQ with respect to δ and θ:


$$\frac{\partial \Delta Q}{\partial \delta }=\frac{uw}{(\theta -1)p}<0$$

$$\frac{\partial \Delta Q}{\partial \theta }=-\frac{(\delta -1)uw}{(\theta -1{)}^{2}p}>0$$

$$\frac{\partial \Delta Q}{\partial r_{B}}=\frac{(1-\theta )uw}{(\theta -1)p}<0$$

Proof of **Proposition 1.**

Let Δπ=0 and solve for $\hat{\delta }$, the critical cost share ratio can be written as follows:


$$\hat{\delta }=\frac{uw(w+p(-1+\theta ))+\sqrt{r_{B}(2uw(-p+w)+2px+uw^{2}r_{B}))-uw^{2}(-1+\theta )(u{\left(p-w\right)}^{2}}}{uw^{2}}$$

Proof of **Proposition 2.**

Let Δπ=0 solve for $\hat{\theta }$, the critical revenue share ratio can be written as follows:


$$\begin{array}{cc} \mathrm{\backslash fontsize}7pt8pt\mathrm{\backslash selectfont}\hat{\theta }= & \frac{1}{2p^{2}u}(\sqrt{r_{B}(uw^{2}r_{B}+2uw(w-p)+2px)+u{\left(p-w\right)}^{2}}\sqrt{r_{B}(uw^{2}r_{B}+2uw(w-p)+2px)+u({\left(p+w\right)}^{2}-4\delta pw)}\\ & +2p(r_{B}(uw-x)+\delta uw)-uw^{2}{\left(r_{B}+1\right)}^{2}+p^{2}u) \end{array}$$

Proof of **Corollary 1.**

We compute the partial derivatives of the profit difference Δπ to the cost share ratio and the revenue share ratio:


$$\frac{\partial \Delta \pi }{\partial \delta }=uw\left(\frac{(\delta -1)w}{(\theta -1)p}-1\right)<0$$

$$\frac{\partial \Delta \pi }{\partial \theta }=\frac{u(p^{2}-\frac{{\left(\delta -1\right)}^{2}w^{2}}{{\left(\theta -1\right)}^{2}})}{2p}$$

Regarding θ, the derivative was evaluated at θ=0 and θ=1:


$${\;\frac{\partial \Delta \pi }{\partial \theta }|}_{\theta =0}=\frac{u(p^{2}-{\left(\delta -1\right)}^{2}w^{2})}{2p}>0$$

$${\;\frac{\partial \Delta \pi }{\partial \theta }|}_{\theta =1}=\frac{up^{2}}{2p}>0$$

Therefore $\frac{\partial \Delta \pi }{\partial \theta }>0$.

Proof of **Proposition 3**

Under normal demand d~N(μ,σ2), the retailer’s profit function with debt financing is:


$$\pi_{R}^{B}=\mu \Phi \left(\frac{q-\mu }{\sigma }\right)+q\left(1-\Phi \left(\frac{q-\mu }{\sigma }\right)\right)-\sigma \phi \left(\frac{q-\mu }{\sigma }\right)-(wq-x)\left(1+r_{D}\right)-x$$

First-Order Condition for Optimal Order Quantity:


$$\frac{\partial \pi_{R}^{B}}{\partial q}=p[\sigma \mu \varphi \left(\frac{q-\mu }{\sigma }\right)+(1-\Phi \left(\frac{q-\mu }{\sigma }\right))-\sigma q\varphi \left(\frac{q-\mu }{\sigma }\right)+\sigma z\varphi \left(\frac{q-\mu }{\sigma }\right)]-w(1+r_{D})$$

Set the derivative to zero for optimality:


$$\Phi \left(\frac{q^{*}-\mu }{\sigma }\right)=1-\frac{\left(1+r_{D}\right)w}{p}$$

Define the implicit function:


$$F(q^{*},\sigma )=\Phi \left(\frac{q^{*}-\mu }{\sigma }\right)-1-\left(\frac{q^{*}-\mu }{\sigma }\right)=0$$

$$\frac{\partial F}{\partial q^{*}}=\Phi \left(\frac{q^{*}-\mu }{\sigma }\right)\frac{1}{\sigma }$$

$$\frac{\partial F}{\partial \sigma }=\varphi \left(\frac{q-\mu }{\sigma }\right)\left(-\frac{q^{*}-\mu }{\sigma^{2}}\right)$$

$$\frac{\partial q^{*}}{\partial \sigma }=-\frac{\frac{\partial F}{\partial \sigma }}{\frac{\partial F}{\partial q^{*}}}=\frac{q^{*}-\mu }{\sigma }$$

The sign of $\;\frac{\partial q^{*}}{\partial \sigma }$ is determined by the relationship between $q^{*}$ and μ.

Proof of **Proposition 4**

Proof of Bank Financing:

Under bank financing, the financing cost is:


$$CB=r_{D}(wq-x)$$

Where $q^{*}=\mu +\sigma \Phi^{-1}(1-\frac{(1+r)w}{p})$. Substituting $q^{*}$:


$$C_{B}=r\left(w\mu +w\sigma \Phi^{-1}\left(1-\frac{(1+r)w}{p}\right)-x\right)$$

$$\frac{\partial C_{B}}{\partial \sigma }=rw\Phi^{-1}\left(1-\frac{(1+r)w}{p}\right)$$

Since p>(1+r)w,$\Phi^{-1}\left(1-\frac{(1+r)w}{p}\right)>0$


$$\frac{\partial C_{B}}{\partial \sigma }>0$$

Proof of RSF:

Under RSF, the financing cost is:


$$C_{R}=\theta pE\left[\min(d,q)\right]$$

Where $q^{*}=\mu +\sigma \Phi^{-1}\left(1-\frac{(1-\delta )w}{(1-\theta )p}\right)$ and $z=\Phi^{-1}\left(1-\frac{(1-\delta )w}{(1-\theta )p}\right).$ Substituting E[min(d,q)]:


$$C_{R}=\theta p\mu +\frac{\theta (1-\delta )\sigma w}{1-\theta }\Phi^{-1}(z)-\theta p\sigma \varphi (z)$$

$$\frac{\partial C_{R}}{\partial \sigma }=\theta p\left(z\left(1-\Phi (z)\right)-\varphi (z)\right)$$

z is between 0 and 1. Therefore z(1−Φ(z))<φ(z) according to Mills’ ratio inequality, therefore:


$$\frac{\partial C_{R}}{\partial \sigma }<0$$

Proof of **Proposition 5.**

Set


$$\Delta =(Q\theta -B-g)(Q(1-\theta )-x-B-\pi_{R}^{D})$$

$$\frac{d\Delta }{d\theta }=Q(-B-\pi_{R}^{D}+(1-\theta )Q-x)-Q(-B-g+\theta Q)$$

$$\frac{d\Delta^{2}}{d\theta^{2}}=-2Q^{2}<0$$

This second derivative indicates that there exists a θ that maximize the Δ

Set $Q\left(-B-\pi_{R}^{D}+(1-\theta )Q-x\right)-Q(-B-g+\theta Q)=0$, find $\theta^{*}$.


$$\theta^{*}=\frac{Q+g-x{-\pi }_{R}^{D}}{2Q}$$

## Supporting information

S1 Data Data and code. (ZIP)

## References

[1] Gelsomino LM, Mangiaracina R, Perego A, Tumino A. Supply chain finance: a literature review. Int J Phys Distrib Logist Manage. 2016;46(4). doi: 10.1108/ijpdlm-08-2014-0173

[2] Jing B, Seidmann A. Finance sourcing in a supply chain. Decision Support Syst. 2014;58:15–20. doi: 10.1016/j.dss.2013.01.013

[3] Kouvelis P, Zhao W. Who Should Finance the Supply Chain? Impact of Credit Ratings on Supply Chain Decisions. M&SOM. 2018;20(1):19–35. doi: 10.1287/msom.2017.0669

[4] Marak ZR, Pillai D. Factors, Outcome, and the Solutions of Supply Chain Finance: Review and the Future Directions. JRFM. 2018;12(1):3. doi: 10.3390/jrfm12010003

[5] Xu X, Chen X, Jia F, Brown S, Gong Y, Xu Y. Supply chain finance: A systematic literature review and bibliometric analysis. Int J Product Econ. 2018;204:160–73. doi: 10.1016/j.ijpe.2018.08.003

[6] Wang J, Zhao L, Huchzermeier A. Operations‐Finance Interface in Risk Management: Research Evolution and Opportunities. Product Operat Manage. 2021;30(2):355–89. doi: 10.1111/poms.13269

[7] Pasternack BA. Commentary—Optimal Pricing and Return Policies for Perishable Commodities. Market Sci. 2008;27(1):131–2. doi: 10.1287/mksc.1070.0347

[8] Roy K. Is Revenue-Based Financing a Risky Business Move? 2024. Available from: https://goviceversa.com/revenue-based-financing-pros-and-cons-risk/.

[9] Cachon GP. Supply Chain Coordination with Contracts. Supply Chain Management: Design, Coordination and Operation. Handbooks in Operations Research and Management Science; 2003. p. 227–339.

[10] Cachon GP, Lariviere MA. Supply Chain Coordination with Revenue-Sharing Contracts: Strengths and Limitations. Management Science. 2005;51(1):30–44. doi: 10.1287/mnsc.1040.0215

[11] Wang YH, Pan JM. Quantity flexibility contracts with unreliable supply. I C Serv Syst Serv M. 2009;:343–6.

[12] Wong WK, Qi J, Leung SYS. Coordinating supply chains with sales rebate contracts and vendor-managed inventory. Int J Product Econ. 2009;120(1):151–61. doi: 10.1016/j.ijpe.2008.07.025

[13] Nie T, Du S. Dual-fairness supply chain with quantity discount contracts. Europ J Operat Res. 2017;258(2):491–500. doi: 10.1016/j.ejor.2016.08.051

[14] Du S, Nie T, Chu C, Yu Y. Newsvendor model for a dyadic supply chain with Nash bargaining fairness concerns. Int J Product Res. 2014;52(17):5070–85. doi: 10.1080/00207543.2014.895446

[15] Dada M, Hu Q. Financing newsvendor inventory. Operations Research Letters. 2008;36(5):569–73. doi: 10.1016/j.orl.2008.06.004

[16] Kouvelis P, Zhao W. Financing the Newsvendor: Supplier vs. Bank, and the Structure of Optimal Trade Credit Contracts. Operat Res. 2012;60(3):566–80. doi: 10.1287/opre.1120.1040

[17] Zhang C, Wang Y, Zhang L. Risk-averse preferences in a dual-channel supply chain with trade credit and demand uncertainty. RAIRO-Oper Res. 2021;55:S2879–903. doi: 10.1051/ro/2020128

[18] Cheng Y, Wu DD, Olson DL, Dolgui A. Financing the newsvendor with preferential credit: bank vs. manufacturer. International Journal of Production Research. 2020;59(14):4228–47. doi: 10.1080/00207543.2020.1759839

[19] Shi J, Du Q, Lin F, Li Y, Bai L, Fung RYK, et al. Coordinating the supply chain finance system with buyback contract: A capital-constrained newsvendor problem. Computers & Industrial Engineering. 2020;146:106587. doi: 10.1016/j.cie.2020.106587

[20] Shen B, Wang X, Cao Y, Li Q. Financing decisions in supply chains with a capital‐constrained manufacturer: competition and risk. Int Trans Operational Res. 2019;27(5):2422–48. doi: 10.1111/itor.12670

[21] Huang J, Yang W, Tu Y. Supplier credit guarantee loan in supply chain with financial constraint and bargaining. Int J Product Res. 2019;57(22):7158–73. doi: 10.1080/00207543.2019.1581386

[22] Xue M, Wu D, Hu H. Pricing and financing strategies of dual channel closed-loop supply chain considering capital-constrained manufacturer. EJIE. 2024;18(2):275–301. doi: 10.1504/ejie.2024.137035

[23] Huang J, Yang W, Tu Y. Financing mode decision in a supply chain with financial constraint. Int J Product Econ. 2020;220:107441. doi: 10.1016/j.ijpe.2019.07.014

[24] Sun S, Hua S, Liu Z. Navigating default risk in supply chain finance: Guidelines based on trade credit and equity vendor financing. Transport Res Part E: Logist Transport Rev. 2024;182:103410. doi: 10.1016/j.tre.2023.103410

[25] Jing B, Chen X, Cai GS. Equilibrium Financing in a Distribution Channel with Capital Constraint. Product Operati Manage. 2012;21(6):1090–101. doi: 10.1111/j.1937-5956.2012.01328.x

[26] Yan N, He X, Liu Y. Financing the capital-constrained supply chain with loss aversion: Supplier finance vs. supplier investment. Omega. 2019;88:162–78. doi: 10.1016/j.omega.2018.08.003

